# Humeral elevation workspace during daily life of adults with spinal cord injury who use a manual wheelchair compared to age and sex matched able-bodied controls

**DOI:** 10.1371/journal.pone.0248978

**Published:** 2021-04-23

**Authors:** Brianna M. Goodwin, Stephen M. Cain, Meegan G. Van Straaten, Emma Fortune, Omid Jahanian, Melissa M. B. Morrow

**Affiliations:** 1 Division of Health Care Delivery Research, Mayo Clinic, Rochester, MN, United States of America; 2 Robert D. and Patricia E. Kern Center for the Science of Health Care Delivery, Mayo Clinic, Rochester, MN, United States of America; 3 Department of Mechanical Engineering, University of Michigan, Ann Arbor, MI, United States of America; 4 Department of Physical Medicine and Rehabilitation, Mayo Clinic, Rochester, MN, United States of America; University of Illinois at Urbana-Champaign, UNITED STATES

## Abstract

Shoulder pain and pathology are extremely common for individuals with spinal cord injuries (SCI) who use manual wheelchairs (MWC). Although risky humeral kinematics have been measured during wheelchair-based activities performed in the lab, little is known about arm kinematics in the free-living environment. The purpose of this study was to measure the humeral elevation workspace throughout a typical day for individuals with SCI who use a MWC and matched able-bodied controls. Thirty-four individuals with SCI who use a MWC (42.7±12.7 years of age, 28 males/6 females, C6-L1) and 34 age-and sex-matched controls were enrolled. Participants wore three inertial measurement units (IMU) on their upper arms and torso for one to two days. Humeral elevation angles were estimated and the percentage of time individuals spent in five elevation bins (0–30°, 30–60°, 60–90°, 90–120°, and 120–180°) were calculated. For both arms, the SCI cohort spent a significantly lower percentage of the day in 0–30° of humeral elevation (Dominant: SCI = 15.7±12.6%, Control = 32.1±15.6%, p<0.0001; Non-Dominant: SCI = 21.9±17.8%, Control = 34.3±15.5%, p = 0.001) and a significantly higher percentage of time in elevations associated with tendon compression (30–60° of humeral elevation, Dominant: SCI = 62.8±14.4%, Control = 49.9.1±13.0%, p<0.0001; Non-Dominant: SCI = 58.8±14.9%, Control = 48.3±13.6%, p = 0.003) than controls. The increased percentage of time individuals with SCI spent in elevations associated with tendon compression may contribute to increased shoulder pathology. Characterizing the humeral elevation workspace utilized throughout a typical day may help in understanding the increased prevalence of shoulder pain and pathology in individuals with SCI who use MWCs.

## Introduction

Shoulder pain is the most common site of musculoskeletal pain in adults with spinal cord injuries (SCI) who use manual wheelchairs (MWC) and its existence can significantly limit a person’s functional abilities [1]. Shoulder pain is reported in 37–70% of individuals with SCI who use a MWC [2–7]. This differs vastly from the 2.9% of the general able-bodied population who experience shoulder pain [8]. Although shoulder pain can develop any time after SCI, it is most commonly developed within the first five years [9] and often lasts longer than one year [3]. Of the MWC users who experience pain, up to 93% have pathological signs on MRI [10], most commonly in the supraspinatus tendon [11].

In general, non-traumatic supraspinatus tendon tears in the shoulder have been thought to be caused by a combination of intrinsic and extrinsic factors [12]. However, these effects can be exacerbated by overuse [13]. One extrinsic factor is the narrowing of the subacromial space which causes compression of the supraspinatus tendons under the coracoacomial arch, and is hypothesized to lead to increased tendon pathology and pain [14,15]. Individualized musculoskeletal models utilizing MRI have estimated the risk of supraspinatus tendon compression through various humeral planes and elevations [16]. The magnitude of glenohumeral elevation was the greatest kinematic predictor of tendon compression risk, followed by the specific plane of elevation. The supraspinatus tendon had the greatest risk of compression at humerothoracic elevations angles between 30–60° [15]. Biplane fluoroscopic imaging of the shoulder joint during dynamic motion has shown similar results and demonstrated that at higher humeral elevations, as the humeral head rotates posteriorly, the supraspinatus tendon may no longer be under the coracoacromial arch, therefore, not at risk of compression [17]. Understanding where tendon compression risk occurs can provide insights when interpreting the humeral elevation workspace of activities of daily living.

MWC propulsion, transfers, and other wheelchair-based activities of daily living have been investigated in laboratory environments to characterize the upper extremity kinematics that pose a risk for shoulder tendon compression from a reduction in subacromial space [18–20]. Although in-laboratory data provide accurate quantifications of how MWC users utilize their arms to complete specific activities, it is unable to quantify the exposure to postures known to reduce subacromial space in daily-living. To understand the daily exposure to shoulder motion and potential shoulder tendon compression, inertial measurement units (IMUs) can be used to measure the angular velocity and acceleration of body segments throughout an entire day in environments of daily living. IMU-based methods for quantifying shoulder movement show good agreement with position-based motion capture and have been used to quantify shoulder elevation angles; however, a limited number of studies have applied these methods to free-living full-day collections [21–26]. To the best of our knowledge no study has utilized these methods to understand the humeral elevation workspace of MWC users throughout an entire day.

The purpose of this study was to use IMUs to measure the humeral elevation workspace throughout a typical day for individuals with SCI who use a MWC and compare it to matched able-bodied controls. Comparison to controls allows for understanding of how humeral elevation exposure during daily life differs when the option to use the lower extremities for weight bearing and mobility is removed. This study also aimed to understand the effects of years of MWC use, pain, sex, and level of SCI on the humeral elevation workspace. Due to the increased prevalence of shoulder pain and pathology in MWC users compared to able-bodied controls [11] and the potential role that humeral elevation has on shoulder tendon compression [15], we hypothesized that MWC users would utilize a different humeral elevation workspace than able-bodied adults. Specifically, we hypothesized individuals with SCI would spend a higher percentage of time at elevation angles previously associated with tendon compression risk [27]. Understanding the humeral elevation workspace of individuals with SCI may contribute to understanding why increased levels of shoulder pain and pathology occur for this population.

## Methods

### Participant enrollment

This study was approved by the Mayo Clinic Institutional Review Board. Individuals with an SCI who used a MWC as their main mode of mobility were recruited through querying medical records and care providers of local clinics. Sex- and age- (±2.5 years) matched able-bodied controls were recruited through email distribution lists and classified ads. Participants for both cohorts were considered for inclusion in the study if they were between 18–70 years of age and had functional range of motion at both shoulders. Functional range of motion was defined as active humeral thoracic flexion, abduction of at least 150° and the ability of the participant to touch the opposite shoulder, the back of his/her neck and his/her low back. Prior to accrual to the study a licensed physical therapist performed a screening physical exam to confirm inclusion and exclusion criteria listed above. This study is part of a larger longitudinal study that follows rotator cuff pathology progression over time via magnetic resonance imaging (MRI). Therefore, participants were also excluded if they self-reported a previous diagnosis of complete supraspinatus tendon tear or they were withdrawn from the study if a complete tear was seen during the first MRI. Participants with SCI who had unilateral supraspinatus complete tears were still eligible to be followed for the contralateral shoulder. Additionally, participants in both cohorts were excluded if there were conditions/factors which might have hindered protocol adherence and controls were also excluded if they had any musculoskeletal or neurological disorder which might have impacted shoulder health or changed the individual’s ability to walk independently.

### Questionnaires and IMU instrumentation

Upon enrollment, participants attended an in-lab visit. A licensed physical therapist screened participants for eligibility and informed consent was obtained. Participants self-reported their hand dominance and were asked if they had pain in either or both shoulders. To assess the presence of shoulder pain, the physical therapist asked the participant if they experience any shoulder pain in either or both shoulders during their daily life. The therapist clarified with the participant that the pain can come and go, is muscular or joint pain and not nerve pain, and may happen before or after certain activities. All participants from both cohorts completed the Disabilities of the Arm, Shoulder, and Hand (DASH) questionnaire [28] for both right and left arms. The DASH is a measure of physical function and symptoms, and is not specific to the shoulder, but rather the whole arm is considered when responding to the questions. It encompasses 30 questions which ask individuals to rate their difficulty, pain, and satisfaction when accomplishing specific tasks on a 5 point scale. Scores range from 0–100, with 0 indicating no difficulty and 100 indicating the most difficulty, pain, and dissatisfaction. The DASH has been shown to be reliable and to have high validity [29]. Additionally, the SCI cohort filled out the Wheelchair User’s Shoulder Pain Index (WUSPI) for both the right and left shoulders. To complete the WUSPI, participants were asked to rate their shoulder pain when completing 15 tasks on a visual analog scale between “no pain” and “worst pain ever experienced” [30]. Possible scores ranged from 0 (no pain) and 150 (worst pain ever experienced in all categories). The WUSPI is valid and reliable for this population [31]. Although we acknowledge that the DASH and WUSPI were designed to be filled out once, as part of a larger study, both surveys were filled out for both arms to evaluate pain and function as it related to each arm.

**Fig 1:**
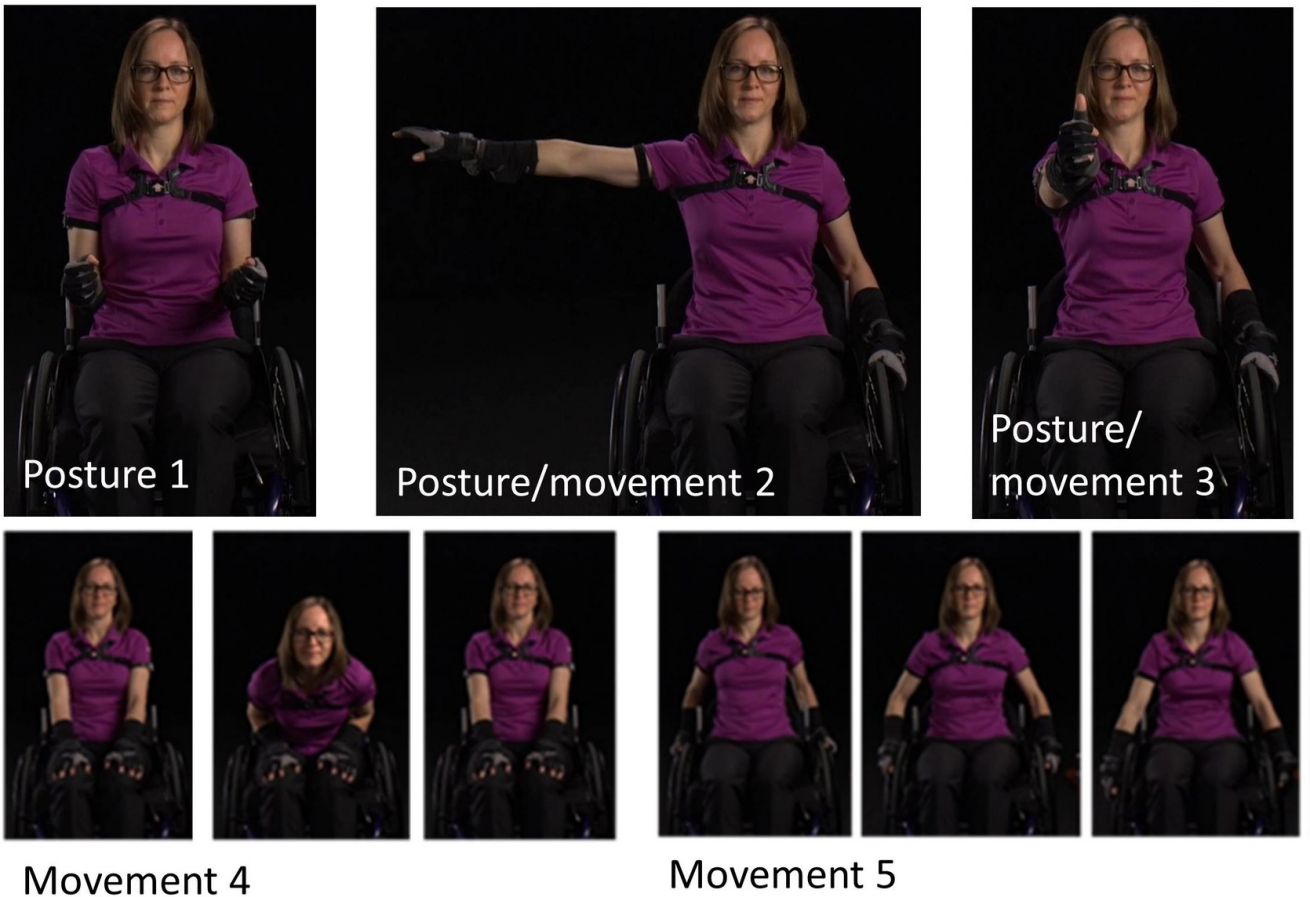
Functional calibration used to align IMU’s with the body. Postures included static upright neutral posture with upper arms resting against the thorax (posture 1), static and dynamic arm t-pose/movement (shoulder abduction = 90°, posture 2), static and dynamic flexion pose/movement (shoulder flexion = 90°, posture 3), dynamic flexion and extension of the torso (movement 4), and simulated wheelchair use or walking (movement 5). Postures 2 and 3 were completed for both the right and left arms separately. (Note: The individual pictured is a co-author who is able-bodied).

Participants were given three wireless IMUs (Emerald or Opal, APDM, Inc., Portland, OR). Each IMU contained a 3-axis accelerometer (±200 g), 3-axis gyroscope (±2000°/s), and 3-axis magnetometer (±8 Gauss). The three IMUs remained synchronized via a proprietary wireless protocol, recorded data at 128 Hz and saved the data to internal storage. In order to maximize the consistency of IMU placement and functional calibration movements across participants, written handouts, video guides and in-person instruction were provided. Participants were instructed to wear one IMU on each lateral upper arm and one on the anterior of the torso; IMUs were secured on the body with elastic and Velcro straps. Each IMU was labeled with the wear location (left arm, right arm, or torso) and an arrow indicating the proper mounting orientation. Participants were instructed to wear the sensors during the entire length of two typical days, excluding bathing and swimming, and take them off before going to bed. Both cohorts were asked to perform their regular daily routines; participants in the control cohort did not use MWCs. Upon donning the sensors for a day, participants performed a set of functional calibration postures (Fig 1, Appendix A, the individual in this manuscript has given written informed consent (as outlined in PLOS consent form) to publish these case details). Due to the collection of multiple days of data, participants were responsible for charging the IMUs overnight using a provided charging station. After the data collection, participants returned the sensors with a pre-paid mailer or in person to the study staff.

### Data processing

**Fig 2:**
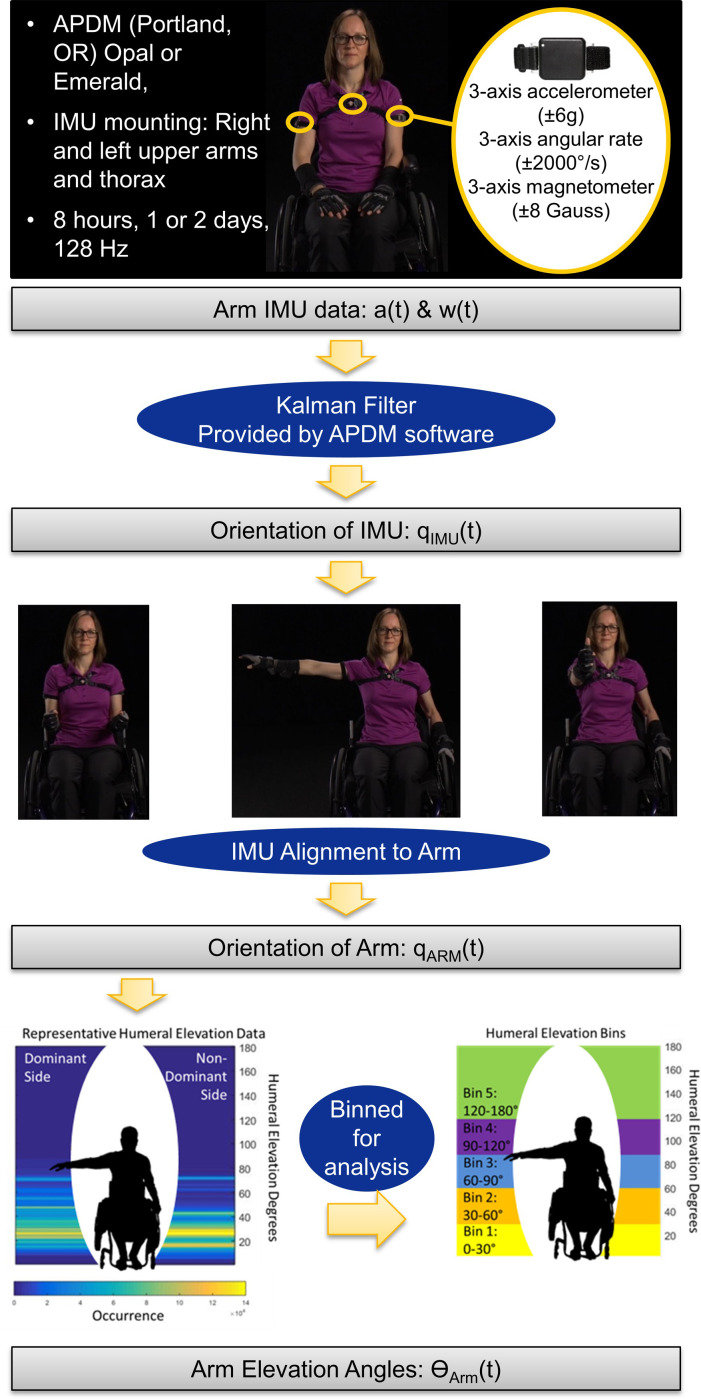
Data processing workflow. This included data collected, IMU orientation, IMU alignment to arm through calibration postures, representative data, and humeral elevation bins. The percentage of time spent in each bin was calculated and used for analysis. (Note: The individual pictured is a co-author who is able-bodied).

Data were downloaded through Motion Studio (APDM, Inc., Portland, OR) and outputs included estimates of the orientations of each IMU relative to an inertial frame (Fig 2). The orientation estimates were derived from the combined acceleration and angular velocity data, rather than only the acceleration data. While researchers have used IMU-measured acceleration only to estimate arm orientation [32,33], there are known limitations to this approach [34], namely, the challenge of separating the measured acceleration into gravitational and body caused components. Algorithms that use both measured acceleration and angular velocity to estimate IMU orientation or attitude (orientation relative to gravity) are well understood and are critical in strapdown inertial navigation [35]. These algorithms integrate the angular velocity signal to estimate orientation during periods with high dynamics (significant body acceleration) and use the acceleration signal to update or correct the orientation during periods with low dynamics (measured acceleration close to the acceleration of gravity). Further, these algorithms take different forms and have been proven to be highly accurate for estimating attitude [36–39]. Additionally, the orientation estimates were calculated without magnetometer data due to the unknown and likely non-uniform magnetic fields present throughout field data collections. While the orientation algorithm used by APDM is proprietary, sensor fusion methods (e.g. Kalman filters) used to estimate IMU orientation from raw sensor data are well understood and well documented in the literature [21,35,36]. Custom MATLAB (Mathworks, Natick, MA) code was written to calculate orientations of anatomical axes relative to IMU-fixed reference frames using data collecting during each participant’s functional calibration postures and movements (Fig 1; Appendix A). Orientation of a given body segment (upper arm or thorax) in an inertial (world) reference frame was then estimated using the orientation of the IMU and the orientation of the anatomical axes relative to the IMU-fixed reference frame (Appendix B). Humeral elevation and thorax deviation angles were defined as the angle between the long axis of the body segment (defined from the function calibration) and vertical; these angles are only dependent on the estimated direction of gravity relative to the body segment and, therefore, are drift-free metrics for quantifying body segment motions. The calculated humeral elevation angles range between 0–180°, with 0° indicating the arm was down and perfectly aligned with gravity and 180° indicating the arm was raised overhead and aligned with gravity. These methods have previously been validated in unpublished data where five individuals with SCI performed 10 reaching tasks. The absolute error and percent of error when compared to the gold standard (electromagnetic system) were -0.06±1.12° and -1.44±1.28%, respectively, for the range of motion. The absolute error and percent of error for the maximum elevation achieved during each reach were 2.59±2.47° and 2.04±2.47%, respectively.

It is important to note that humerothoracic elevation angles and elevation planes relative to the thorax were not calculated as these calculations require accounting for relative drift between the orientation estimates of arm and torso IMUs. While the attitude estimates are accurate and do not drift, the yaw or heading estimates, which describe the rotation angle or direction of a body segment about a vertical axis, do drift, making accurate calculation of humerothoraic angles over long periods of time difficult [24]. This difficulty is best illustrated by the fact that studies that use IMUs to quantify shoulder motion during long periods in the real world either do not calculate shoulder angles [32,40–42] or acknowledge the limitations of the methodology [24]. Other work [25,43] claims to accurately calculate shoulder angle of elevation but not plane of elevation; however, shoulder angle of elevation cannot be calculated accurately without the plane of elevation [44]. Correcting the drift between sensors about vertical is an active research area and requires a joint specific approach [45,46]. Therefore, in our analysis, data in which the thorax deviation angle was more than 30° were eliminated in order to allow humeral elevation angles to be interpreted similarly to humerothoracic elevation angles; 30° was selected based on an unpublished sensitivity analysis performed during a prior study.

The percentage of daily wear time each participant spent in five humeral elevation bins were calculated (0–30°, 30–60°, 60–90°, 90–120°, and 120–180°). The bin sizes were chosen as a means to combine three theories: 1) a painful arc of motion occurs between 60–120° of arm abduction [47], 2) Rapid Upper Limb Assessment (RULA) which bins risky arm postures between 0–20°, 20–45°, 45–90°, and >90° [48], and 3) the subacromial risk area of 30–60° [15].

Periods of non-wear were determined using methods from Lugade and colleagues (2014) [49] and were excluded from data analysis. Data were also excluded from analysis if the functional calibration postures were not completed properly or if at least eight hours of data were not collected after the elimination of non-wear time. Data were included if one or two complete days were collected; if two days were included all data were combined before the calculation of the percent of time in humeral elevation bins.

### Statistical analysis

Between cohort differences for the demographics data were assessed using paired t-tests for the continuous variables (weight, height, and DASH), McNemar’s test for the presence of shoulder pain and Fisher’s Exact test for hand dominance. Multivariate analyses of variance (MANOVA) were used to test for the main effect of cohort on time spent in each humeral elevation bin of both the dominant and non-dominant side (α = 0.05). Similarly, within each cohort, MANOVA was also used to test main effects of sex, age, and arm function (DASH) on time in bins. Finally, withing the MWC user cohort, MANOVA was used to test main effects of shoulder pain (WUSPI), level of SCI, and years of MWC use. Linear regression analysis was used to test the strength of the relationship between the time spent in each humeral elevation bin with age and years of MWC use for the MWC cohort. Within cohorts, analysis of variance (ANOVA) was used to test the effect of humeral elevation bin for both arms. When significant main effects were observed, post hoc paired t-tests were performed. A Bonferroni correction factor was used to adjust the alpha level from 0.05 to 0.01 due to comparisons across five bins.

## Results

Thirty-four participants with SCI who used a MWC, and 34 age (±2.5 years) and sex matched, able-bodied adults were enrolled (Table 1). There were no statistical differences between the cohort’s self-reported weight, height, and dominant hand.

**Table 1:** Participant demographics.

	SCI	Control	P-value
**Sample size**	34	34	-
**Age**	42.7 +/- 12.7 (22.6–63.3)	42.6 +/- 12.5 (24.3–61.0)	
**Sex**	28 males/6 females	28 males/6 females	-
**Self-reported weight (kg)**	80.7 +/- 17.2 (54.0–145.1)	81.6 +/- 17.5 (56.7–149.7)	0.822
**Self-reported height (cm)**	177.4 +/- 7.6 (160.0–195.6)	178.4 +/- 9.5 (160.0–205.7)	0.417
**Injury Level**			
Cervical (C6-C8)	7	-	-
High/mid thoracic (T1-T8)	16		
Low thoracic/lumbar (T9-L1)	11		
**Years of manual wheelchair use (years)**	11.5 +/- 10.7 (0.5–36.0)	-	-
**Dominant arm**	27 right/7 left	32 right/2 left	0.374
**DASH (dominant arm)**	15.2 +/- 17.5 (0–71.7)	1.3 +/- 2.9 (0–15)	<0.0001
**DASH (non-dominant arm)**	13.6 +/- 14.2 (0–51.7)	1.1 +/- 3.3 (0–15)	<0.0001
**WUSPI (dominant arm)**	12.7 +/- 20.4 (0–71.2)	-	-
**WUSPI (non-dominant arm)**	10.8 +/- 16.2 (0–71.6)	-	-
**Self-reported shoulder pain (Number of participants reporting pain, % of cohort)**	26 (76%)	9 (26%)	<0.0001

### Excluded data

**Fig 3:**
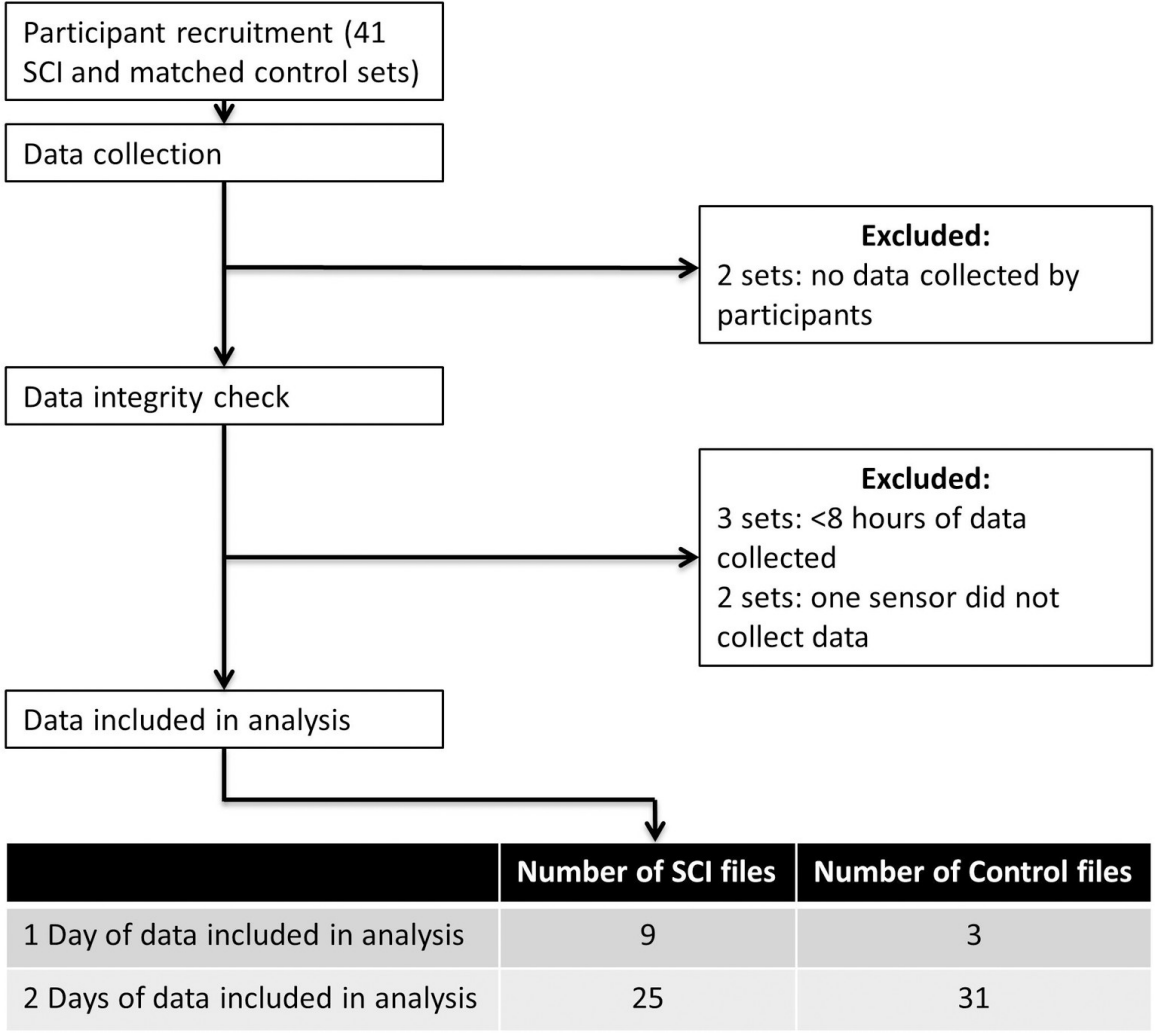
Data exclusion processes. Data were excluded if either SCI or control did not collect data, a minimum of 8 hours of data were not collected, or one sensor malfunctioned. Data were included in analysis if one or two days of data were collected.

One control participant was ineligible for the study due to a self-reported complete supraspinatus tear which was confirmed with the medical records of an MRI. Seven pairs of data were excluded from the analysis due to exclusion criteria (Fig 3). Data were collected for an average (SD) of 11.4(2.1) and 11.9(1.3) hours for the SCI and control cohorts, respectively. Additionally, on average 18.3(14.0) and 28.0(10.3) percent of the day was excluded because the trunk was at or over 30° for the SCI and control cohorts, respectively.

### Humeral elevation workspace

**Fig 4:**
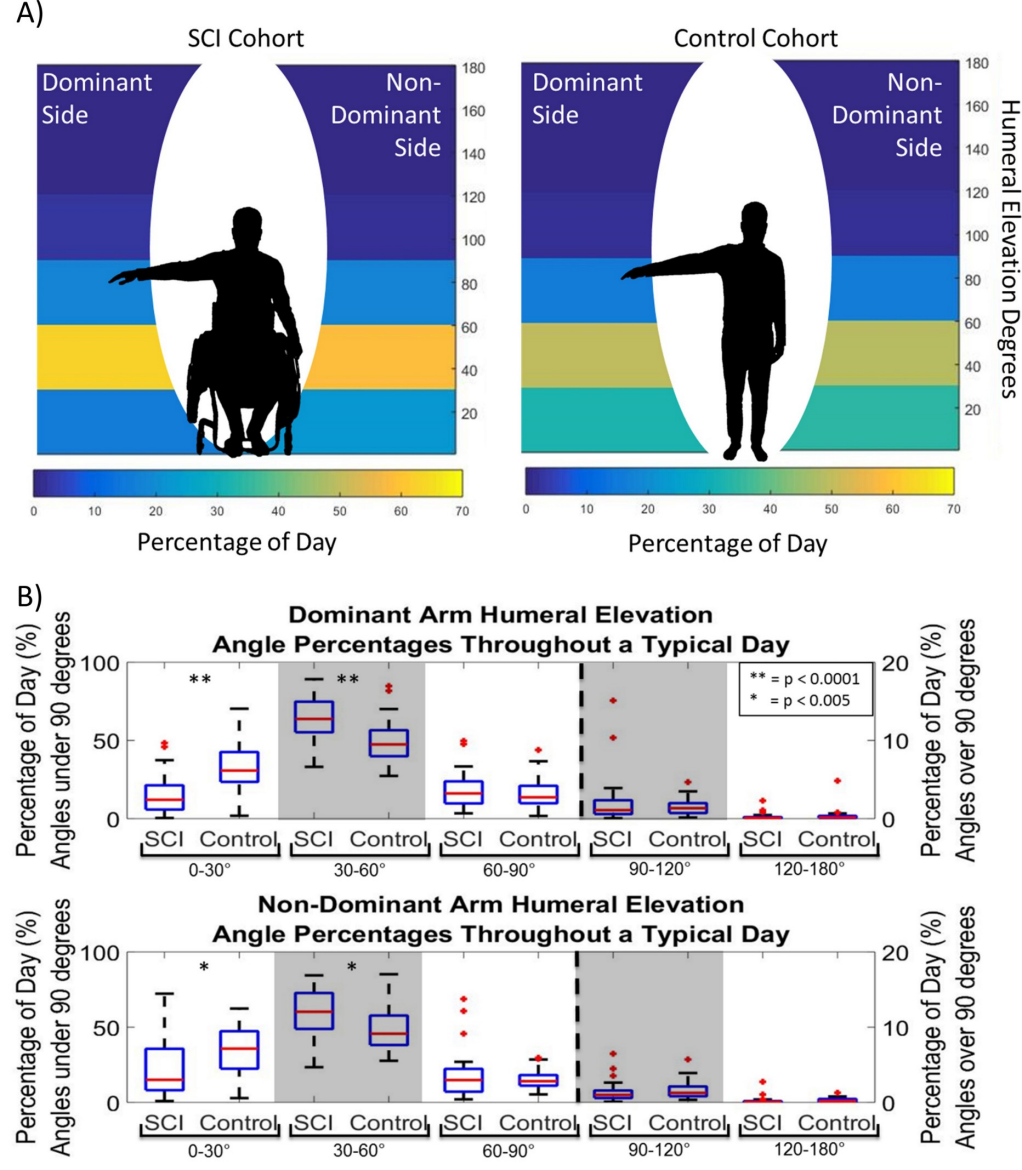
The percentage of time in each humeral elevation. A) The average percentage of time throughout a typical day individuals in the SCI and control cohorts spent in 0–30°, 30–60°, 60–90°, 90–120°, and 120–180° of humeral elevation for their dominant and non-dominant sides. B) Percentage of time throughout a typical day individuals in the SCI and control cohorts spent in 0–30°, 30–60°, 60–90°, 90–120°, and 120–180° of humeral elevation for their dominant arm (top) and their non-dominant arm (bottom). For each boxplot the central line (red) represents the median, the edges of the box are the 25^th^ and 75^th^ percentiles, and the error bars extend the most extreme data points not considered outliers and, the outliers are denoted by red +. ** indicates p < 0.0001 and * indicates p < 0.005.

There was a main effect of cohort across humeral elevation bins on both dominant and non-dominant sides (p<0.0001). Additionally, there was a main effect of humeral elevation bin for both cohorts and arms (dominant: p<0.0001, non-dominant: p = 0.005, Fig 4). Individuals with SCI spent significantly more time in 30–60° of humeral elevation than all other elevations bins on both their dominant and non-dominant sides (p<0.001, Table 2). The SCI cohort spent 63% and 59% of their daily wear time (approximately 7 hours per day) at these elevations on their dominant and non-dominant sides, respectively. The controls also spent the greatest amount of daily wear time in this elevation bin at 50% and 48% on their dominant and non-dominant arm respectively, which was significantly lower than the SCI cohort for both arms (dominant: p < .0001, non-dominant: p = 0.003, Table 2).

**Table 2:** The average (SD) percentage of the day individuals with SCI and matched able-bodied controls spent in five humeral elevation bins throughout one or two days.

Bin	SCI Dominant Arm Percentage (%)	Control Dominant Arm Percentage (%)	P-Value	SCI Non-Dominant Arm Percentage (%)	Control Non-Dominant Arm Percentage (%)	P-Value
**0–30°**	15.7 (12.6)	32.1 (15.6)	<0.0001	21.9 (17.8)	34.3 (15.5)	0.001
**30–60°**	62.8 (14.4)	49.9 (13.0)	<0.0001	58.8 (14.9)	48.3 (13.6)	0.003
**60–90°**	18.4 (11.0)	16.2 (9.6)	0.410	17.7 (14.8)	15.6 (6.2)	0.430
**90–120°**	2.8 (5.3)	1.4 (1.0)	0.145	1.4 (1.4)	1.6 (1.2)	0.589
**120°-180°**	0.2 (0.4)	0.4 (0.8)	0.320	0.2 (0.5)	0.3 (0.3)	0.430

For the SCI cohort, the second largest percentage of time was spent in 60–90° of humeral elevation (approximately 20% of their day for both arms). Controls spent their second largest percentage of time in 0–30° of elevation for both arms, which was significantly higher than the amount of time the SCI cohort spent in this elevation bin (p<0.001). Individuals with SCI spent comparable amounts of time in 0–30° and 60–90° of elevation, while controls spent significantly more time in 0–30° than 60–90° of humeral elevation on the dominant (p<0.001) and non-dominant (p<0.0001) sides.

On average, participants in both cohorts spent less than 3% of their day (<25 minutes) in elevations over 90° for both arms. There were no significant differences between cohorts for the 60–90° 90–120°and >120° humeral elevation bins or between dominant and non-dominant arms for each cohort and each elevation bin.

### Pain and arm function

Pain, measured by the WUSPI (in the SCI cohort) and arm function measured by the DASH (in both both cohorts), did not have a significant effect on the percentage of time an individual spent in any humeral elevation bins for both dominant and non-dominant arms.

### Sex, age, injury level, and years of MWC use

There were no main effects of sex (Table 3), age (Table 4), injury level (Table 3), or years of MWC Use (Table 4) on either arm.

**Table 3:** The percentage of time individuals spent in humeral elevation bins based on their injury level and sex.

**Injury Level**						
	Cervical		High/mid thoracic		Low thoracic/lumbar	P-Value
Dominant Arm (% of the day)						
**0–30°**	20.3 ± 12.5		15.5 ± 14.0		13.1 ± 11.4	0.521
**30–60°**	57.4 ± 11.0		63.0 ± 15.9		66.0 ± 15.1	0.493
**60–90°**	18.0 ± 9.7		18.4 ± 12.4		18.8 ± 11.2	0.988
**90–120°**	3.7 ± 5.2		3.0 ± 6.9		1.8 ± 2.9	0.769
**120–180°**	0.6 ± 0.8		0.1 ± 0.2		0.2 ± 0.2	0.050
Non-Dominant Arm (% of the day)						
**0–30°**	24.7 ± 16.9		20.6 ± 21.1		22.1 ± 15.0	0.888
**30–60°**	56.9 ± 11.3		59.4 ± 18.6		59.0 ± 12.6	0.937
**60–90°**	16.5 ± 9.1		18.9 ± 19.		16.6 ± 11.4	0.910
**90–120°**	1.6 ± 1.2		1.0 ± 1.0		1.9 ± 1.9	0.275
**120–180°**	0.3 ± 0.4		0.06 ± 0.09		0.4 ± 0.8	0.266
**Sex**						
		Male		Female		P—Value
Dominant Arm (% of the day)						
**0–30°**		14.4 ± 12.		21.9 ± 15.3		0.199
**30–60°**		62.8 ± 14.4		63.2 ± 17.4		0.950
**60–90°**		19.4 ± 11.7		13.8 ± 8.0		0.267
**90–120°**		3.2 ± 5.9		1.0 ± 0.8		0.381
**120–180°**		0.2 ± 0.5		0.2 ± 0.3		0.814
Non-Dominant Arm (% of the day)						
**0–30°**		20.4 ± 18.3		29.0 ± 16.2		0.294
**30–60°**		59.1 ± 15.6		57.4 ± 14.2		0.814
**60–90°**		18.8 ± 16.1		12.2 ± 7.6		0.337
**90–120°**		1.5 ± 1.5		1.2 ± 0.8		0.691
**120–180°**		0.2 ± 0.5		0.1 ± 0.1		0.565

**Table 4:** Linear regression results for the percentage of time individuals spent in humeral elevation bins based on age and years of MWC use.

	**Age**		**Years of MWC use**	
	R^2^	P-Value	R^2^	P-Value
Dominant Arm				
**0–30°**	0.104	0.06	0.009	0.60
**30–60°**	0.037	0.28	0.000	0.97
**60–90°**	0.013	0.41	0.008	0.62
**90–120°**	0.000	0.94	0.002	0.82
**120–180°**	0.002	0.79	0.047	0.22
Non-Dominant Arm				
**0–30°**	0.027	0.35	0.001	0.84
**30–60°**	0.002	0.81	0.009	0.58
**60–90°**	0.025	0.37	0.007	0.65
**90–120°**	0.000	0.88	0.054	0.19
**120–180°**	0.049	0.21	0.023	0.39

## Discussion

This study aimed to understand the humeral elevation workspace utilized throughout a typical day by individuals with SCI who use a MWC. These results were compared to a matched able-bodied control cohort to better understand factors which may contributed to a higher rate of both pain and tendon pathology associated with years of MWC use [11]. Both individuals with SCI and controls spent the majority of their day (~80%) in elevation angles between 0 and 60°. However, individuals with SCI spent significantly more time in humeral elevations previously found to be associated with supraspinatus tendon compression (30–60°) than controls [27,50,51]. There was no evidence of the effect of injury level, years of MWC use, age, or sex on the humeral elevation workspace for individuals with SCI.

With the growing capabilities of wearable technology, many SCI-specific algorithms have been created and validated to accompany and enhance data captured in a lab setting [52]. Many of the studies using wearable technology to understand movement of MWC users have focused specifically on wheelchair propulsion and use [53,54], with less focus on understanding humeral elevation angles or overuse of the arms of MWC users. The data presented in the current study supplements data collected in a laboratory setting and other free-living MWC use metrics by providing lengths of exposure to risky postures in the free-living environment.

Recently it has been suggested that compression of the supraspinatus tendon occurs at low elevation angles. Giphart, et al. [17] suggested that subacromial impingement syndrome occurs below 70° of humeral elevation and the minimum distance between the footprint of the supraspinatus tendon and greater tuberosity occurred between 36° and 65° of humeral elevation during forward flexion. Additionally, using individualized bone models (from MRI) and group averaged kinematics, Lawrence, et al. [15] used musculoskeletal simulation models to suggest the minimum distance between the coracoacomial arch and supraspinatus tendon area occurred at 42° of humerothoracic elevation. Our results show that individuals with SCI who use a MWC spent significantly more time than controls in a similar range of humeral elevations (30–60°). This difference could be in part due to differences in the arm elevation workspace during mobility. During MWC propulsion the humeral elevation is approximately 25 to 55° at a self-selected speed [55,56]; however, during walking, the humeral elevation angles required are much lower [57]. The difference in humeral elevation during mobility likely is not the only contributor to this increase, as MWC users move about 3 km less than able-bodied individuals and only spend a small amount of their day actually propelling themselves; estimates range from 16 to 54 minutes per day [53,58]. Another contributing factor to this discrepancy may be wheelchair setup; for example, MWC users may not place their arms in a neutral resting position of 0–30° due to the location of their arm rest. It will be important in future studies to understand whether a large proportion of the time in 30–60° of humeral elevation is actually due to MWC users resting on their arm rests. In addition to the humeral elevation workspace differing during propulsion for MWC users and walking for able-bodied individuals, the loading of the shoulder is also different during these two tasks and likely contributes to the increase in pathology in MWC users. Further, additional data collections and analyses are needed to fully understand the clinical implications of the differences in humeral elevation between the wheelchair users and able-bodied control group.

Capturing a holistic view of an individual’s exposure to potentially risky humeral elevation is dependent on many factors including occupation and activities performed throughout a day. A study looking at 10 able-bodied elderly adults using only accelerometry data found that less than 4% of an individual’s day was spent in elevations above 90°, with the average elevation angle occurring at 40° [43]. These results are very similar to the data presented in the current study for both cohorts; about 3% of the day was spent in elevations over 90°. Previous reports have suggested that extended periods of time in overhead motion may be the cause of increased shoulder pain. Our results paired with the most recent modeling and imaging data may suggest that injury to the supraspinatus tendon due to tendon compression of the SCI cohort also occurs in-part due to increased time between 30–60° of humeral elevations. Further, pain in higher elevation angles may be caused by other mechanisms [15]. Continuing to map this workspace for individuals with SCI who use a MWC while they perform specific tasks (i.e., propulsion or transfers) may help us to further understand daily risk exposures and the contribution of specific tasks.

Multiple challenges exist when using unsupervised real-world IMU data. First, accounting for and correcting the drift of IMU-based body segment orientation estimates is a common challenge in understanding the relative orientation of body segments (i.e. joint angles), especially for extended data collections (see excellent discussion in [24]). The current algorithms utilized in this study do not take the plane of motion into account; 30° of humeral elevation in front of the body, to the side, or behind would all be interpreted as 30° of humeral elevation and are indistinguishable. While we could have used the orientation estimates to calculate humerothoracic angle of elevation and plane of elevation, we know that those calculations would contain errors from the heading/yaw drift. Heading drift directly affects the plane of elevation and accurately calculating the plane of elevation is critical to accurately calculating humerothoracic angle of elevation. Therefore, the data presented here only used the angle of the humerus relative to vertical (humeral elevation angle) and not the trunk (humerothoracic angle). This was compensated for by eliminating humeral elevation time points where the trunk angle was at or over 30° of tilt; participants may have been leaning over or lying down. On average about 10% more data were eliminated from the control data sets than the SCI data sites, indicating the controls had more variability and movement of their trunk than the SCI cohort. Even with these limitations, the methods used in this study to estimate sensor orientation and humeral elevation are more accurate than other methods using only acceleration data, especially during movements with high dynamics [36–39].

Since this study included a limited number of participants with a cervical level SCI who use manual wheelchairs, these results should not be generalized to individuals with a cervical level SCI who use power wheelchairs. Power wheelchairs often have the ability to recline or tilt in space. Individuals with a higher level cervical SCI may also routinely sit in a more reclined position to increase their stability and compensate for lack of trunk control. If persons with higher level cervical SCIs perform a large amount of arm movement in these reclined or tilted positions, then a large portion of their daily routine data would be eliminated by the 30° trunk tilt threshold that was used in this study. If this is the case, a different approach to study arm use in this population would be needed.

There are limitations with the data presented in this study to consider. Previous studies have found that up to four days of data collection are needed to represent propulsion trends consistent throughout a MWC user’s daily life [59]. Only one or two days of data were collected for participants in this study due to participant availability and adherence to the protocol. We attempted to compensate for this by asking participants to wear the sensors on ‘typical days;’ however, we did not account for the day of the week or whether it was a workday or not in our analysis. Further, our analysis represents the data from the full day and does not account for the distribution of the humeral elevation angles at specific segments of the day such as the morning, afternoon or evening. The calibration protocol used in this study enabled us to determine humeral elevations without an in-lab calibration. As participants performed the calibration protocol unsupervised, it’s possible that there could be errors induced by incorrect neutral and 90° calibration postures. The data presented here were binned into 30° ranges below 120° of humeral elevation; however, creating bins with different boundaries may affect the results. Appendix C shows the average percent of time in 10° bins. Additionally, there are other factors beyond humeral elevation that contribute to shoulder injury in the SCI population including scapular motion, shoulder muscle strength, and increased load on the shoulder due to MWC propulsion, body transfers, and repetitive motion. Loading of the shoulder although not measured in this study, has an important role in the increased pathology and pain for MWC users. This analysis does not report rotator cuff pathology and how it relates to differences in daily humeral elevations between the cohorts. Shoulder tendon pathology from MRI is part of a larger longitudinal study that follows rotator cuff pathology via MRI over time, and future reports will provide meaningful information about humeral elevations and associations with pathology progression.

## Conclusions

This study aimed to understand the humeral elevation workspace throughout a typical day of individuals with SCI who use a MWC and compare it to the workspace of age- and sex-matched controls. Our data suggest that individuals with SCI who use a MWC may spend more time in a potentially risky humeral elevation range (30–60°) than the controls. The findings from this study do not support an effect of age, sex, pain, injury level, or years since injury on the humeral elevation workspace for adults with SCI who use a MWC. Future work should expand the understanding of loading of the upper extremity during daily life and characterize more in-depth information about shoulder workspace and activities of daily living across injury levels and groups with and without pain and pathology.

## Supporting information

S1 Appendix Defining sensor-to-segment alignment matrices. (DOCX)

S2 Appendix Calculating humeral elevation thorax deviation angles. (DOCX)

S3 Appendix The distribution of the percentage of time the SCI and control cohort spent in 10° bins for both the dominant and non-dominant arms. (TIF)

S4 Appendix Data underlying the humeral elevation plots for each participant in each humeral elevation bin for dominant (dom) and nondominant (nondom) arms. (S) indicates SCI participant and (C) indicates control participant.
(XLSX)
