# Supplementary material for: Humeral elevation workspace during daily life of adults with spinal cord injury who use a manual wheelchair compared to age and sex matched able-bodied controls
Source: PLoS One. 2021 Apr 23;16(4):e0248978. doi: 10.1371/journal.pone.0248978 (PMC8064589; doi:10.1371/journal.pone.0248978)
Supplement: S1 Appendix — (DOCX) [file pone.0248978.s001.docx]

**Appendix A:** Defining sensor-to-segment alignment matrices

When using inertial sensors to quantify the movement of body segments, determining the sensor-to-segment alignment is critical to enable accurate quantification of the orientation of the body segment rather than the orientation of the sensor. In other words, the orientation of a body segment’s anatomical reference frame with respect to the sensor reference frame must be determined so that estimates of the sensor orientation can be used to understand body segment orientation. In our study, we utilize functional alignment movements and postures, in which participants complete some known movements or poses for which at least one anatomical axis can be estimated in an inertial measurement unit’s (IMU’s) body-fixed frame of reference.

Our functional alignment poses and movements are as follows:

1. Sitting/standing upright with arms at sides
2. Right arm t-pose (Figure 1 Posture 2)
3. Dynamic ab/adduction of the right shoulder, keeping right arm movement in frontal plane
4. Right arm flexion pose (Figure 1 Posture 3)
5. Dynamic flexion/extension of the right shoulder, keeping right arm movement in sagittal plane
6. Left arm t-pose (similar to Figure 1 Posture 2)
7. Dynamic ab/adduction of the left shoulder, keeping left arm movement in frontal plane
8. Left arm flexion pose (similar Figure 1 Posture 3)
9. Dynamic flexion/extension of the left shoulder, keeping left arm movement in sagittal plane
10. Dynamic trunk flexion/extension, keeping movement of thorax in sagittal plane
11. Sitting/standing upright with arms at sides
12. Simulated wheelchair propulsion or arm swing

Using data (IMU measured accelerations and angular velocities) from the above poses and movements, we construct sensor-to-segment alignment matrices for the thorax, right arm, and left arm as described below.

For the thorax, we first use the average acceleration due to gravity measured by the torso-mounted IMU during FA1 and FA11 to define a body segment fixed z- axis (superior-inferior axis,$\hat{Z}_{thorax}$) for the thorax:

|  | $\hat{Z}_{thorax}=\frac{a_{FA1 \& FA11}}{\sqrt{a_{FA1 \& FA11}\cdot a_{FA1 \& FA11}}}$ |  |
| --- | --- | --- |

This establishes the thorax posture for which the thorax deviation angle is equal to zero. The average axis of rotation for the thorax (torso-mounted IMU) measured during FA10 is used to define the thorax x-axis (medial-lateral axis) for the thorax. To determine the average axis of rotation of the thorax segment ($\hat{X}_{thorax}$), a principal components analysis is performed on the measured segment angular velocity measured during FA10. The first principal component is the unit vector defining the average axis of rotation.

We define an anterior-posterior axis ($\hat{Y}_{thorax}$) for the thorax as the unit vector orthogonal to the superior-inferior axis and medial-lateral axis:

|  | $\hat{Y}_{thorax}=\frac{\hat{Z}_{thorax}\times\hat{X}_{thorax}}{\mathrm{norm}\left( \hat{Z}_{thorax}\times\hat{X}_{thorax} \right)}$ |  |
| --- | --- | --- |

Finally, we ensure that the medial-lateral axis is orthogonal to the anterior-posterior and superior-inferior axes:

|  | $\hat{X}_{thorax}=\hat{Y}_{thorax}\times\hat{Z}_{thorax}$ |  |
| --- | --- | --- |

The resulting unit vectors ($\hat{X}_{thorax},\hat{Y}_{thorax},\hat{Z}_{thorax}$) define a body-segment fixed frame aligned with estimated anatomical axes. The direction cosine matrix that defines the transformation of measurements made in the sensor-fixed frame to those in a thorax-fixed frame is defined by Equation (4), where each row of the matrix contains the components of the segment-fixed axes.

|  | $R_{thorax\vert sensor}=\left[ \begin{matrix} \hat{X}_{thorax} \\ \hat{Y}_{thorax} \\ \hat{Z}_{thorax} \end{matrix} \right]$ |  |
| --- | --- | --- |

For the left and right arm, we aim to establish an axis that represents the long-axis of the humerus (defined here as the z-axis). Starting with the right arm, we first get one estimate of the z-axis of the upper arm by using acceleration measured during FA2 and angular velocity measured during FA3.

|  | $\hat{X}_{RArm1}=\frac{a_{FA2}}{\sqrt{a_{FA2}\cdot a_{FA2}}}$ |  |
| --- | --- | --- |

A principal components analysis is performed on the measured segment angular velocity measured during FA3; the first principal component is the unit vector defining the average axis of rotation during FA3 ($\hat{Y}_{RArm1}$). The first estimate of the z-axis of the upper arm is calculated:

|  | $\hat{Z}_{RArm1}=\hat{X}_{RArm1}\times\hat{Y}_{RArm1}$ |  |
| --- | --- | --- |

We choose $\hat{Z}_{RArm1}= \hat{Z}_{RArm1}$ or $\hat{Z}_{RArm1}=-\hat{Z}_{RArm1}$ such that $\hat{Z}_{RArm1}$ points superiorly (determined by examining $a_{FA1}$ for the right arm). We get a second estimate of the z-axis of the upper arm by using acceleration measured during FA4 and angular velocity measured during FA5.

|  | $\hat{X}_{RArm2}=\frac{a_{FA4}}{\sqrt{a_{FA4}\cdot a_{FA4}}}$ |  |
| --- | --- | --- |

A principal components analysis is performed on the measured segment angular velocity measured during FA5; the first principal component is the unit vector defining the average axis of rotation during FA5 ($\hat{Y}_{RArm2}$). The second estimate of the z-axis of the upper arm is calculated:

|  | $\hat{Z}_{RArm2}=\hat{X}_{RArm2}\times\hat{Y}_{RArm2}$ |  |
| --- | --- | --- |

We choose $\hat{Z}_{RArm2}= \hat{Z}_{RArm2}$ or $\hat{Z}_{RArm2}=-\hat{Z}_{RArm2}$ such that $\hat{Z}_{RArm2}$ points superiorly (determined by examining $a_{FA1}$ for the right arm).

We define the right arm z-axis using the two estimates:

|  | $\hat{Z}_{RArm}=\frac{mean(\left[ \hat{Z}_{RArm1};\hat{Z}_{RArm2} \right])}{norm(mean\left( \left[ \hat{Z}_{RArm1};\hat{Z}_{RArm2} \right] \right))}$ |  |
| --- | --- | --- |

We construct a right arm x-axis:

|  | $\hat{X}_{RArm}=\frac{\hat{Y}_{RArm1}\times\hat{Z}_{RArm}}{\mathrm{norm}\left( \hat{Y}_{RArm1}\times\hat{Z}_{RArm} \right)}$ |  |
| --- | --- | --- |

We choose $\hat{X}_{RArm}= \hat{X}_{RArm}$ or $\hat{X}_{RArm}=-\hat{X}_{RArm}$ such that $\hat{X}_{RArm}$ points up during FA2 (determined by examining $a_{FA2}$ for the right arm).

Finally, we construct a right arm y-axis:

|  | $\hat{Y}_{RArm}=\hat{Z}_{RArm}\times\hat{X}_{RArm}$ |  |
| --- | --- | --- |

The resulting unit vectors ($\hat{X}_{RArm},\hat{Y}_{RArm},\hat{Z}_{RArm}$) define a body-segment fixed frame aligned with estimated anatomical axes for the right arm. The direction cosine matrix that defines the transformation of measurements made in the sensor-fixed frame to those in a right arm-fixed frame is defined by Equation (12), where each row of the matrix contains the components of the segment-fixed axes.

|  | $R_{RArm\vert sensor}=\left[ \begin{matrix} \hat{X}_{RArm} \\ \hat{Y}_{RArm} \\ \hat{Z}_{RArm} \end{matrix} \right]$ |  |
| --- | --- | --- |

For the left arm we first get one estimate of the z-axis of the upper arm by using acceleration measured during FA6 and angular velocity measured during FA7.

|  | $\hat{X}_{LArm1}=\frac{a_{FA6}}{\sqrt{a_{FA6}\cdot a_{FA6}}}$ |  |
| --- | --- | --- |

A principal components analysis is performed on the measured segment angular velocity measured during FA7; the first principal component is the unit vector defining the average axis of rotation during FA7 ($\hat{Y}_{LArm1}$). The first estimate of the z-axis of the upper arm is calculated:

|  | $\hat{Z}_{LArm1}=\hat{X}_{LArm1}\times\hat{Y}_{LArm1}$ |  |
| --- | --- | --- |

We choose $\hat{Z}_{LArm1}= \hat{Z}_{LArm1}$ or $\hat{Z}_{LArm1}=-\hat{Z}_{LArm1}$ such that $\hat{Z}_{LArm1}$ points superiorly (determined by examining $a_{FA1}$ for the left arm). We get a second estimate of the z-axis of the upper arm by using acceleration measured during FA8 and angular velocity measured during FA9.

|  | $\hat{X}_{LArm2}=\frac{a_{FA8}}{\sqrt{a_{FA8}\cdot a_{FA8}}}$ |  |
| --- | --- | --- |

A principal components analysis is performed on the measured segment angular velocity measured during FA9; the first principal component is the unit vector defining the average axis of rotation during FA9 ($\hat{Y}_{LArm2}$). The second estimate of the z-axis of the upper arm is calculated:

|  | $\hat{Z}_{LArm2}=\hat{X}_{LArm2}\times\hat{Y}_{LArm2}$ |  |
| --- | --- | --- |

We choose $\hat{Z}_{LArm2}= \hat{Z}_{LArm2}$ or $\hat{Z}_{LArm2}=-\hat{Z}_{LArm2}$ such that $\hat{Z}_{LArm2}$ points superiorly (determined by examining $a_{FA1}$ for the left arm).

We define the left arm z-axis using the two estimates:

|  | $\hat{Z}_{LArm}=\frac{mean(\left[ \hat{Z}_{LArm1};\hat{Z}_{LArm2} \right])}{norm(mean\left( \left[ \hat{Z}_{LArm1};\hat{Z}_{LArm2} \right] \right))}$ |  |
| --- | --- | --- |

We construct a left arm x-axis:

|  | $\hat{X}_{LArm}=\frac{\hat{Y}_{LArm1}\times\hat{Z}_{LArm}}{\mathrm{norm}\left( \hat{Y}_{LArm1}\times\hat{Z}_{LArm} \right)}$ |  |
| --- | --- | --- |

We choose $\hat{X}_{LArm}= \hat{X}_{LArm}$ or $\hat{X}_{LArm}=-\hat{X}_{LArm}$ such that $\hat{X}_{LArm}$ points down during FA6 (determined by examining $a_{FA6}$ for the left arm).

Finally, we construct a left arm y-axis:

|  | $\hat{Y}_{LArm}=\hat{Z}_{LArm}\times\hat{X}_{LArm}$ |  |
| --- | --- | --- |

The resulting unit vectors ($\hat{X}_{LArm},\hat{Y}_{LArm},\hat{Z}_{LArm}$) define a body-segment fixed frame aligned with estimated anatomical axes for the left arm. The direction cosine matrix that defines the transformation of measurements made in the sensor-fixed frame to those in a left arm-fixed frame is defined by Equation (19), where each row of the matrix contains the components of the segment-fixed axes.

|  | $R_{LArm\vert sensor}=\left[ \begin{matrix} \hat{X}_{LArm} \\ \hat{Y}_{LArm} \\ \hat{Z}_{LArm} \end{matrix} \right]$ |  |
| --- | --- | --- |
