# Supplementary material for: Humeral elevation workspace during daily life of adults with spinal cord injury who use a manual wheelchair compared to age and sex matched able-bodied controls
Source: PLoS One. 2021 Apr 23;16(4):e0248978. doi: 10.1371/journal.pone.0248978 (PMC8064589; doi:10.1371/journal.pone.0248978)
Supplement: S2 Appendix — (DOCX) [file pone.0248978.s002.docx]

**Appendix B:** Calculating humeral elevation thorax deviation angles

The humeral elevation angle and thorax deviation angle are defined as the angle between the segment z-axis (defined in Appendix A) and vertical and are calculated by first calculating the direction cosine matrix describing the orientation of the body segment in the inertial/world reference frame:

|  | $R_{world\vert segment}=R_{world\vert sensor}\left( R_{segment\vert sensor} \right)^{T}$ |  |
| --- | --- | --- |

Where $R_{world|sensor}$ is the direction cosine matrix describing the rotation from the sensor reference frame to the inertial/world reference frame and $R_{sensor|segment}$ is the sensor-to-segment alignment from Equation (4), (12), or (20). Humeral elevation angles and thorax deviation angles are then calculated from Equation (22).

|  | $\theta=acos\left( R_{world\vert segment}\left( 3,: \right)\cdot\left[ \begin{matrix} 0 & 0 & 1 \end{matrix} \right] \right)$ |  |
| --- | --- | --- |

Where $R_{world|segment}\left( 3,: \right)$ refers to the last row of $R_{world|segment}$.
