# Supplementary figures and images for: Humeral elevation workspace during daily life of adults with spinal cord injury who use a manual wheelchair compared to age and sex matched able-bodied controls
Source: PLoS One. 2021 Apr 23;16(4):e0248978. doi: 10.1371/journal.pone.0248978 (PMC8064589; doi:10.1371/journal.pone.0248978)

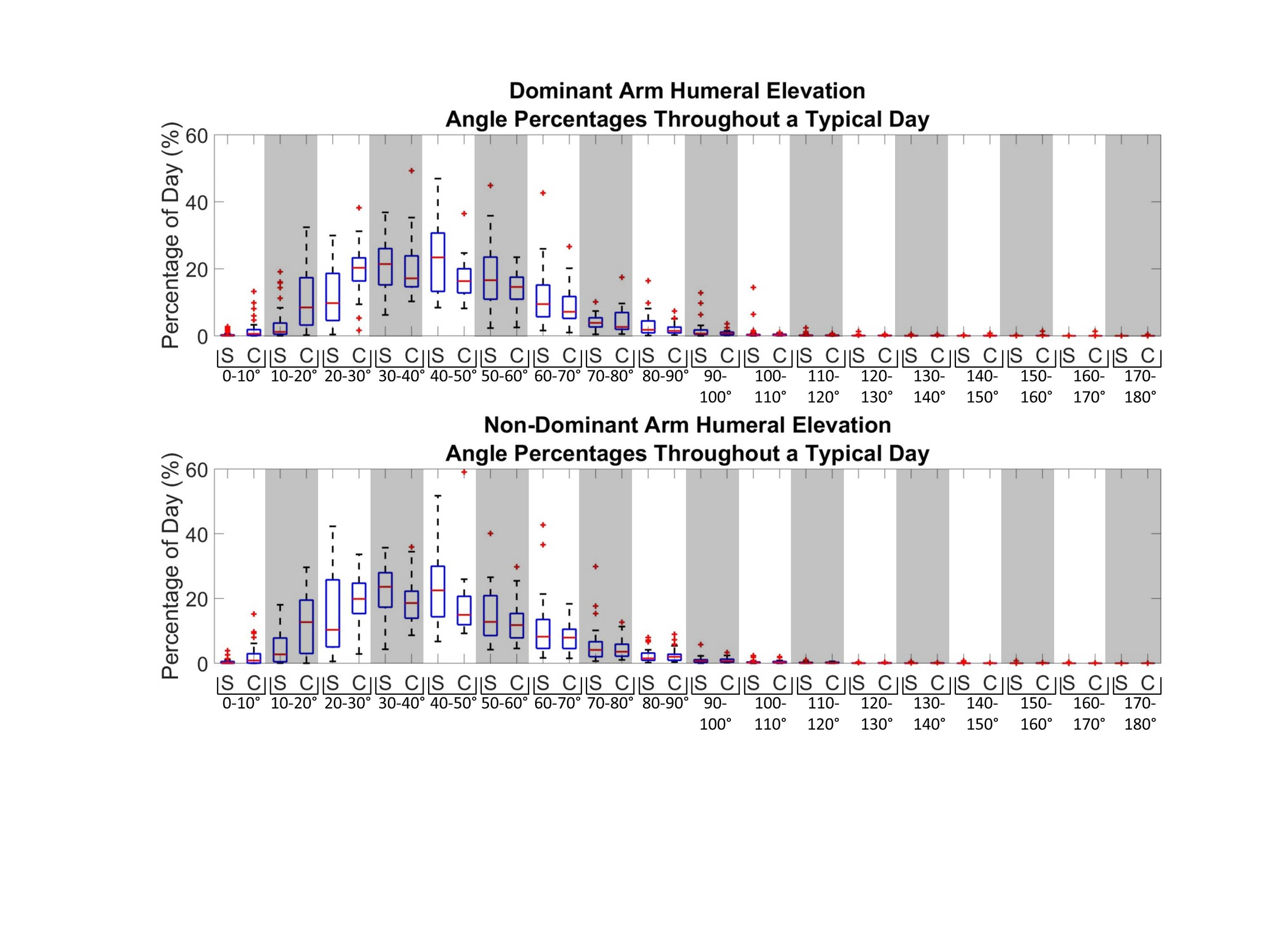

Supplement: S3 Appendix — (TIF) [file pone.0248978.s003.tif]
